# Sensory Nerve Maintains Intervertebral Disc Extracellular Matrix Homeostasis Via CGRP/CHSY1 Axis

**DOI:** 10.1002/advs.202202620

**Published:** 2022-09-01

**Authors:** Bo Hu, Xiao Lv, Leixin Wei, Yunhao Wang, Genjiang Zheng, Chen Yang, Fazhi Zang, Jianxi Wang, Jing Li, Xiaodong Wu, Zhihao Yue, Qiangqiang Xiao, Zengwu Shao, Wen Yuan, Jinsong Li, Peng Cao, Chen Xu, Huajiang Chen

**Affiliations:** ^1^ Spine Center Department of Orthopedics Changzheng Hospital Naval Medical University Shanghai 200003 China; ^2^ Department of Orthopaedics Union Hospital Tongji Medical College Huazhong University of Science and Technology Wuhan 430022 China; ^3^ State Key Laboratory of Cell Biology Shanghai Key Laboratory of Molecular Andrology CAS Center for Excellence in Molecular Cell Science Institute of Biochemistry and Cell Biology Chinese Academy of Science Shanghai 200031 China

**Keywords:** chondroitin sulfate, chondroitin sulfate synthase 1, intervertebral disc degeneration, nucleus pulposus, sensory nerves

## Abstract

Sensory nerves are long being recognized as collecting units of various outer stimuli; recent advances indicate that the sensory nerve also plays pivotal roles in maintaining organ homeostasis. Here, this study shows that sensory nerve orchestrates intervertebral disc (IVD) homeostasis by regulating its extracellular matrix (ECM) metabolism. Specifically, genetical sensory denervation of IVD results in loss of IVD water preserve molecule chondroitin sulfate (CS), the reduction of CS bio‐synthesis gene chondroitin sulfate synthase 1 (CHSY1) expression, and dysregulated ECM homeostasis of IVD. Particularly, knockdown of sensory neuros calcitonin gene‐related peptide (CGRP) expression induces similar ECM metabolic disorder compared to sensory nerve denervation model, and this effect is abolished in CHSY1 knockout mice. Furthermore, in vitro evidence shows that CGRP regulates nucleus pulposus cell CHSY1 expression and CS synthesis via CGRP receptor component receptor activity‐modifying protein 1 (RAMP1) and cyclic AMP response element‐binding protein (CREB) signaling. Therapeutically, local injection of forskolin significantly attenuates IVD degeneration progression in mouse annulus fibrosus puncture model. Overall, these results indicate that sensory nerve maintains IVD ECM homeostasis via CGRP/CHSY1 axis and promotes IVD repair, and this expands the understanding concerning how IVD links to sensory nerve system, thus shedding light on future development of novel therapeutical strategy to IVD degeneration.

## Graphical abstract

The first evidence which demonstrates how sensory nerves communicate with intervertebral disc (IVD) nucleus pulposus cell to maintain its homeostasis. Particularly, IVD water‐preserving molecule chondroitin sulfate (CS) and CS bio‐synthetic gene chondroitin sulfate synthase 1 (CHSY1) are found to be under control by sensory nerve secretion of calcitonin gene‐related peptide (CGRP) via receptor activity‐modifying protein 1 (RAMP1)/cyclic AMP response element‐binding protein (CREB) signaling; dysregulation of this pathway accelerates IVD degeneration. In addition, therapeutic role of intradiscal forskolin injection is established.

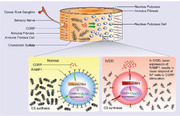

## 1 Introduction

Sensory nerve could sense various outer stimuli, maintain organ metabolic homeostasis, and participate in pain transduction.^[^ ^1^, ^2^, ^3^, ^4^ ^]^ Healthy intervertebral disc (IVD) is a well‐known un‐innervated organ in its nucleus pulposus (NP) region;^[^ ^5^ ^]^ however, sensory nerves are located at vertebrae between IVDs and outer layer of the annulus fibrosus (AF) region of IVD in physiological conditions,^[^ ^5^ ^]^ and these sensory nerve endings are often thought to be involved in low back pain occurrence.^[^ ^5^, ^6^ ^]^ Whether these sensory nerve fibers possess functions other than IVD pain transduction remains unclear. Based on previous studies, other than signal transmission function, sensory nerves also orchestrate body homeostasis and are indispensable in tissue repair, liver regeneration, and bone matrix deposition.^[^ ^7^, ^8^, ^9^ ^]^ Therefore, it is reasonable to speculate that sensory nerve might have functions other than pain transduction in IVD.

Loss of water content of IVD which results in attenuation of IVD integrity is considered a hallmark of IVD degeneration (IVDD). The water abundant feature of IVD relies on the sufficient amount of water preservation molecule chondroitin sulfate (CS) and structure molecule collagen‐II (Col‐II) and aggrecan (ACAN) in NP region, which consists of NP extracellular matrix (ECM).^[^ ^10^ ^]^ The decreased NP ECM anabolism and increased ECM catabolism are considered pivotal IVDD phenotype.^[^ ^10^, ^11^ ^]^ In addition, our previous studies have demonstrated that loss of chondroitin sulfate synthases (CHSYs) expression induced insufficiency of CS content was the major reason that triggered ECM metabolism disorder.^[^ ^12^, ^13^, ^14^ ^]^ However, the upper‐stream regulatory mechanism of CHSYs is still remained elusive.

Recent studies uncovered that sensory neuropeptide could participate in multiple organ physiology and pathophysiology processes such as wound healing, inflammatory responses, and energy metabolism.^[^ ^15^, ^16^, ^17^ ^]^ By mass spectrum analysis, we detected high level of sensory neuropeptide calcitonin gene‐related peptide (CGRP) secretion in mice lumbar dorsal root ganglia (DRG). CGRP was reported to be able to promote osteoblastic bone matrix deposition,^[^ ^18^ ^]^ suggesting its potential pro‐anabolic role. In contrast, we also found genetical denervation of sensory nerve in mice results in significant down regulation of CS bio‐synthesis family gene chondroitin sulfate synthase 1 (CHSY1) in NP cell. Thus, it is possible that sensory nerve secretion of CGRP could regulate NP cell CHSY1 gene to maintain IVD homeostasis.

## 2 Results

### 2.1 Denervation of Sensory Nerve Decreases CS Bio‐Synthesis to Disrupt IVD Homeostasis

**Figure 1:**
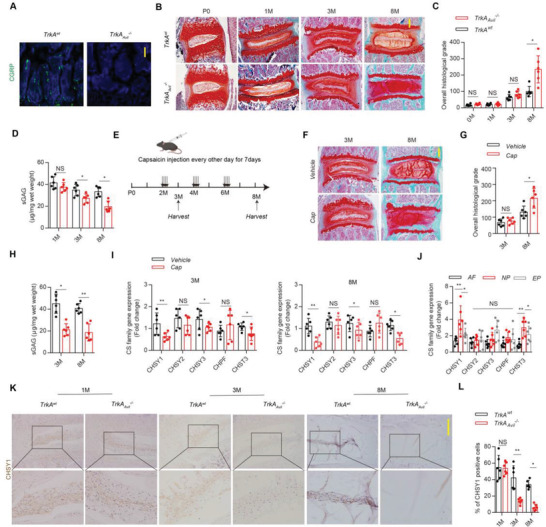
Denervation of sensory nerve decreases CS bio‐synthesis to disrupt IVD homeostasis. A) Representative images of immunofluorescence of CGRP^+^ sensory nerves (green) in the vertebra from 3‐month‐old male *TrkA^wt^* and *TrkA_Avil_^–/–^* mice. Scale bar: 100 µm. B,C) Representative images of Safarin‐O/Fast green staining (SOFG) and quantitative analysis of overall histological grade for *TrkA^wt^* and *TrkA_Avil_^–/–^* mice between postnatal 0 day (P0) to 8‐month (8 M). Scale bar: 50 µm. D) Quantitative analysis of chondroitin sulfate (CS) content by 1,9‐dimethylmethylene blue (DMMB) assay from 1, 3, and 8‐month‐old male *TrkA^wt^* and *TrkA_Avil_^–/–^* mice. E) Schematic graph of the study of capsaicin injection procedure. Male C57B6/J mice were treated with capsaicin 30 mg per kg per day (30 mg/kg/d) subcutaneously every other day for one week at 2‐month‐old, and boosting injections were performed every two months; mice were harvested at 3 and 8‐month‐old. One down arrow represents one time capsaicin injection. F,G) Representative Safarin‐O/Fast green staining images and quantitative analysis of overall histological grade for 3 and 8‐month‐old male C57B6/J mice treated with capsaicin and vehicle. Scale bar: 50 µm. H) Quantitative analysis of CS by DMMB assay from 3 and 8‐month‐old male C57B6/J mice treated with capsaicin and vehicle. I) Quantitative RT‐PCR analysis CS family genes (CHSY1, CHSY2, CHSY3, CHPF, and CHST3) expression of NP tissue from 3 and 8‐month‐old male C57B6/J mice treated with capsaicin and vehicle. J) Quantitative RT‐PCR analysis CS family genes (CHSY1, CHSY2, CHSY3, CHPF, and CHST3) expression of NP, annulus fibrosis (AF), and endplates (EP) in 3‐month‐old WT mice. *P < 0.05 and **P < 0.01, NS: not significant. Statistical significance was determined by one‐way ANOVA. K,L) Representative immunohistochemical staining and quantitative analysis of CHSY1 expression in the NP tissue from 1, 3, and 8‐month‐old male *TrkA^wt^* and *TrkA_Avil_^–/–^* mice. Scale bar: 20 µm. All data are presented as means ± standard error of the mean (SEM), *n* = 6 per group, (C,D,G,H,I,L). *P < 0.05 and **P < 0.01, NS: not significant. Statistical significance was determined by two‐tailed Student's *t*‐test.

To examine the effect of sensory nerve denervation on IVD, we crossed *Advilin‐Cre* mice with nerve growth factor (NGF) receptor *TrkA* floxed (*TrkA^wt^*) mice to generate *TrkA_Avil_^–/–^* mice. Sensory nerve fibers and *TrkA* gene expression were successfully ablated in vertebral area and DRG region, respectively, as evidenced by immunofluorescence (IF) assay (**Figure** **1**A; Figure S1A, Supporting Information). The IVD histological grade and ECM catabolic genes expression were significantly increased, and CS content and ECM anabolic genes were significantly decreased in 8‐month‐old *TrkA_Avil_^–/–^* mice relative to their wild type (WT) littermates as evidenced by safarin O‐fast green (SOFG) staining, real time (RT)‐PCR and 1,9‐dimethylmethylene blue (DMMB) assay (Figure 1A–D; Figure S2A, Supporting Information), while these parameters remained unaffected in 3‐month‐old *TrkA_Avil_^–/–^* mice compared with controls (Figure 1A–D; Figure S2A, Supporting Information), suggesting the impact of sensory nerve to NP ECM homeostasis majorly occurred in mice in late adulthood. Consistently, the above phenotype was also observed in capsaicin (Cap)‐induced sensory denervation mice model (Figure 1E–I; Figure S2B,C, Supporting Information). Concerning the behavior comparability between capsaicin injection model and *TrkA_Avil_^–/–^* mice model, we showed that the capsaicin‐injected mice possessed significantly longer time latency in hot plate, and lower reactions in Von Frey tests, similar to *TrkA_Avil_^–/–^* mice model (Figure S3A,B, Supporting Information). Furthermore, RT‐PCR analysis verified that CS synthase genes CHSY1, 3 and CHST3 expression were significantly lower in NP of adult *TrkA_Avil_^–/–^* mice relative to WT controls, and among these genes, CHSY1 was mostly expressed in NP tissue and the most down‐regulated gene in both 3‐month‐old and 8‐month‐old *TrkA_Avil_^–/–^* mice as determined by RT‐PCR and immunohistochemistry (IHC) assay (Figure 1J–L), suggesting sensory nerve regulation on NP CHSY1 expression might be responsible for its capability of IVD ECM maintenance.

### 2.2 Deletion of CHSY1 Expression Induces IVD ECM Metabolic Disorder in Mice Early Adulthood

**Figure 2:**
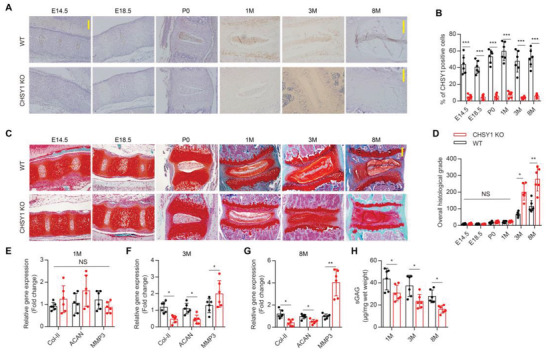
IVD ECM metabolic disorder occurred in early adulthood of CHSY1 KO mice. A,B) Representative immunohistochemical staining and quantitative analysis of CHSY1 expression in the NP for CHSY1 KO and their littermates between embryonic 14.5 day (E14.5) to 8‐month. Scale bar: 50 µm. C,D) Representative images of SOFG and quantitative analysis of overall histological grade for CHSY1 KO and their littermates between embryonic 14.5 day to 8‐month. Scale bar: 50 µm. E,F,G) Quantitative RT‐PCR analysis of NP Col‐II and ACAN and MMP3 expression from 1, 3, and 8‐month‐old male CHSY1 KO and their littermates. H) Quantitative analysis of CS by DMMB assay from 1, 3, and 8‐month‐old male CHSY1 KO and their littermates. All data are presented as means ± SEM, *n* = 6 per group, *P < 0.05, **P < 0.01, and ***P < 0.001, NS: not significant. Statistical significance was determined by two‐tailed Student's *t*‐test.

To further testify the functional role of CHSY1 in IVD ECM metabolism, we generated CHSY1 global knockout (KO) mice by using CRISPR‐Cas9 technology with semi‐cloning technology as previous described (Figure S4A–D, Supporting Information).^[^ ^19^ ^]^ Next, IHC assay verified that CHSY1 expression was successfully deleted in mice embryonic, postnatal stage and adulthood (**Figure** **2**A,B); SOFG staining showed that IVD structural integrity was significantly obstructed in 3‐month‐old and 8‐month‐old CHSY1 KO mice relative to their WT littermates (Figure 2C,D), suggesting congenital loss of CHSY1 expression could efficiently trigger early IVDD progression. Intriguingly, we also found that CHSY1 KO mice possessed lower pain threshold which results in more pain sensitivity compared with their WT littermate as evidenced by Von–Frey and hot‐plate behavior test (Figure S5A,B, Supporting Information). Consistently, RT‐PCR analysis showed that Col‐II and ACAN gene expression were significantly decreased, whereas MMP3 gene expression was significantly increased in 3‐month‐old and 8‐month‐old NP tissue of CHSY1 KO mice compared with controls; however, these genes expressions remained unaffected between 1‐month‐old CHSY1 KO mice and their WT littermates (Figure 2E–G), validating ECM metabolic disorder was induced by CHSY1 KO. Specifically, DMMB assay showed a significantly NP CS content reduction in CHSY1 KO mice relative to their littermates in all 1, 3, and 8‐month‐old mice (Figure 2H).

### 2.3 CHSY1 is Indispensable in Sensory Nerve‐Regulated IVD ECM Homeostasis

**Figure 3:**
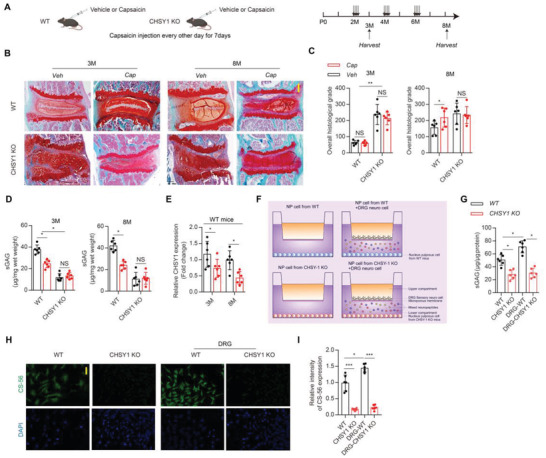
CHSY1 is critical in sensory nerve regulated IVD ECM homeostasis. A) Schematic graph of the study of capsaicin injection procedure on CHSY1 KO. Male CHSY1 KO mice and their littermates were treated with capsaicin (30 mg/kg/d) subcutaneously every other day for one week at 2‐month‐old, and boosting injections were performed every two months; mice were harvested at 3 and 8 months. B,C) Representative images of SOFG and quantitative analysis of overall histological grade for 3 and 8‐month‐old CHSY1 KO mice and their littermates treated with vehicle and capsaicin. Scale bar: 50 µm. D) Quantitative analysis of CS by DMMB assay from 3 and 8‐month‐old male CHSY1 KO mice and their littermates treated with vehicle and capsaicin. E) Quantitative RT‐PCR analysis of NP CHSY1 expression from 3 and 8‐month‐old male CHSY1 KO and their littermates treated with vehicle and capsaicin. F) Schematic graph for in vitro co‐culture system of DRG sensory neuros and NP cells from WT and CHSY1 KO mice. G) Quantitative analysis of CS by DMMB assay for NP cells from CHSY1 KO mice and littermates, which were cultured with DRG neurons or cultured alone. H,I) Representative immunofluorescence staining of CS‐56 (green) and quantitative analysis for NP cells which from CHSY1 KO mice and littermates, were cultured with DRG neurons or cultured alone. Scale bar: 50 µm. All data are presented as means ± SEM, *n* = 6 per group, *P < 0.05, **P < 0.01, and *** *P* < 0.001, NS: not significant. Statistical significance was determined by two‐way repeated measures ANOVA with Bonferroni post hoc test.

To clarify whether CHSY1 is involved in sensory nerve‐regulated NP ECM metabolism, capsaicin induced sensory denervation model was established in WT and CHSY1 KO mice (**Figure** **3**A). Interestingly, we showed that IVDD histological grade and ECM catabolic genes were significantly increased, and CS content and ECM anabolic genes were significantly decreased in 8‐month‐old sensory denervated WT mice, and these effects were abolished both in 3‐month‐old and 8‐month‐old CHSY1 KO mice as evidenced by SOFG staining, RT‐PCR, and DMMB assay (Figure 3B–E; Figure S6A, Supporting Information), suggesting CHSY1 KO ablates sensory nerve regulation on IVD ECM metabolism. Consistently, co‐culturing NP cells with sensory neuros revealed significant increment in NP cells CS production and CHSY1 gene expression compared with NP cells cultured alone, and this effect was obstructed in CHSY1 KO NP cells as evidenced by DMMB, RT‐PCR, western‐blot (WB), and IF assay (Figure 3F–I; Figure S6B,C, Supporting Information), suggesting sensory nerve could affect NP cell CS production via its direct regulation on CHSY1 expression. Surprisingly, we did not observe significant alteration in ECM metabolic genes (Col‐II, ACAN, and MMP3) expression between normal and CHSY1 KO NP cells regardless of their co‐culture status (Figure S6D, Supporting Information), indicating sensory nerve and CHSY1 could not directly affect NP ECM metabolism in vitro.

### 2.4 CGRP is the Major Sensory Neuropeptide Which Responds to CHSY1 KO Induced CS Insufficiency

**Figure 4:**
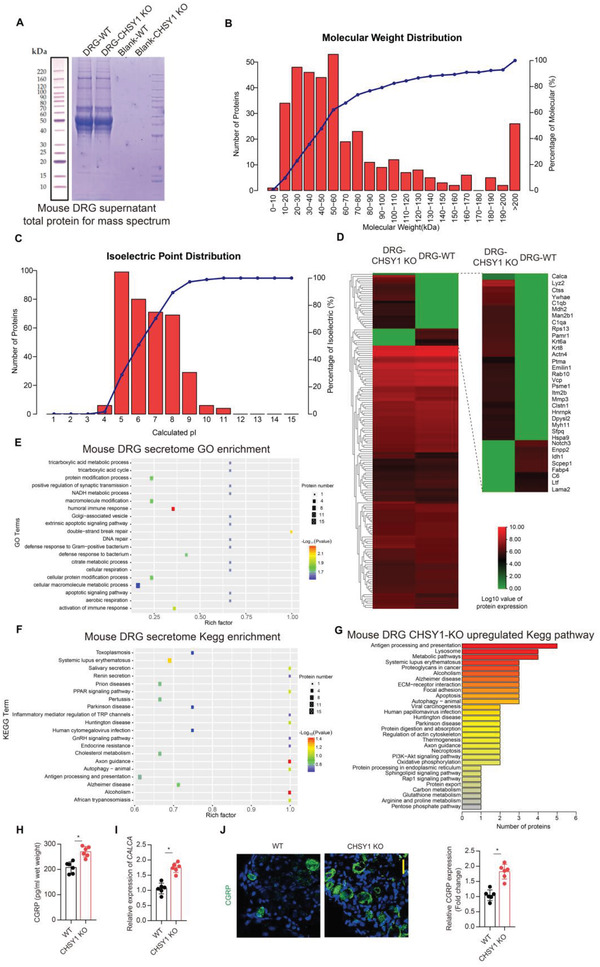
CGRP is the primary DRG neuropeptide which responds to CHSY1 KO‐induced CS insufficiency. A) SDS‐PAGE analysis of total protein expression of mouse DRG cells supernatant from CHSY1 KO and littermates. B) Molecular weight distribution of mouse DRG cells supernatant total protein from CHSY1 KO and littermates. C) Isoelectric point distribution of mouse DRG cells supernatant total protein from CHSY1 KO and littermates. D) Unsupervised hierarchical cluster shows differentially expressed proteins in DRG from CHSY1 KO mice and their littermates. E) The GO enrichment analysis of mouse DRG secretome, differentially expressed proteins mainly related to synaptic transmission and immune response. F,G) The enriched pathway in mouse DRG secretome through Kegg analysis. H) Quantitative analysis of ELISA for CGRP concentration in DRG from CHSY1 KO and their littermates. I) Quantitative RT‐PCR analysis of DRG CALCA gene expression from CHSY1 KO and their littermates. J) Representative immunofluorescence staining of CGRP (green) and quantitative analysis for DRG from CHSY1 KO and their littermates. Scale bar: 10 µm. All data are means ± SEM, *n* = 6 per group, *P < 0.05. Statistical significance was determined by two‐tailed Student's *t*‐test.

To further examine the secretome of mice DRG sensory neuro cells, we performed label‐free proteomic analysis of the supernatant of cultured DRG cells from WT and CHSY1 KO mice (**Figure** **4**A). The protein extracted from the cultured supernatant mainly focused around 10–80 kDa (Figure 4B), and the isoelectric point distribution mainly ranged from 5 to 9 (Figure 4C). The unsupervised hierarchical cluster showed differentially expressed proteins over the two groups (Figure 4D). The gene ontology (GO) analysis (Figure 4E) showed that the differentially expressed proteins were related to synaptic transmission (GO:0050806) and immune response (GO:0002253). Through KEGG analysis (Figure 4F,G), we found that the differentially expressed proteins were related to Axon guidance (KO04360) and endocrine factors related pathways (KO04912, KO04924). Among the main GO and KEGG categories, we found the sensory neuropeptide CGRP secretion by CHSY1 KO DRG cells was significantly increased compared to WT group (Figure 4D). To validate these findings, enzyme‐linked immunosorbent assay (ELISA), RT‐PCR, and IF assay revealed that CGRP secretion and its gene CALCA expression in sensory neuros were significantly up‐regulated in CHSY1 KO group compared to WT group (Figure 4H–J). These pieces of evidence suggest CGRP might be the major response molecule that regulates NP CS synthesis.

### 2.5 Sensory Neuropeptide CGRP Promotes CHSY1 Expression Via RAMP1/CREB Signaling in NP Cells

**Figure 5:**
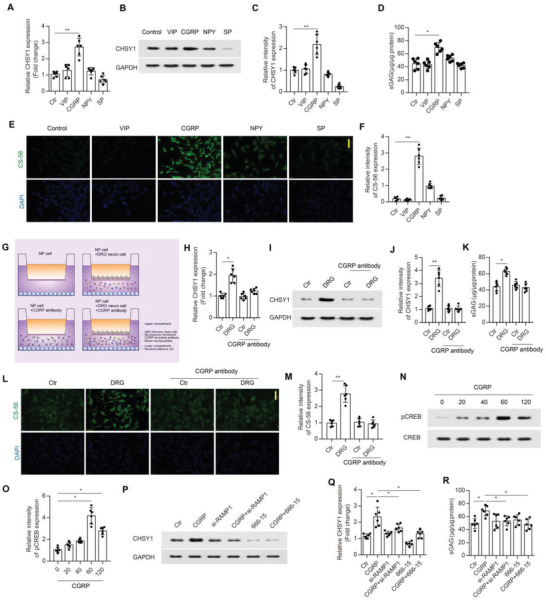
DRG‐derived CGRP promotes CHSY1 expression in NP cell via RAMP1/CREB signaling. A) Quantitative RT‐PCR analysis of CHSY1 expression from NP cells treated with Vehicle, VIP, CGRP, NPY, and SP at the concentration of 10^–7^ m, respectively. B,C) Representative images of WB and quantitative analysis of CHSY1 expression for NP cells treated with Vehicle, VIP, CGRP, NPY, and SP. D) Quantitative analysis of CS by DMMB assay for NP cells treated with Vehicle, VIP, CGRP, NPY, and SP. E,F) Representative immunofluorescence staining of CS‐56 (green) and quantitative analysis for NP cells treated with Vehicle, VIP, CGRP, NPY, and SP. Scale bar: 50 µm. G) Schematic graph for in vitro co‐culture system of DRG sensory neuros and NP cells treated with CGRP neutralizing antibody. H) Quantitative RT‐PCR analysis of CHSY1 expression from NP cells in the DRG co‐culture system treated with CGRP neutralizing antibody and vehicle. I,J) Representative images of WB and quantitative analysis of CHSY1 expression for NP cells in the DRG co‐culture system treated with CGRP neutralizing antibody and vehicle. K) Quantitative analysis of CS by DMMB assay for NP cells in the DRG co‐culture system treated with CGRP neutralizing antibody and vehicle. L,M) Representative immunofluorescence staining of CS‐56 (green) and quantitative analysis for NP cells in the DRG co‐culture system treated with CGRP neutralizing antibody and vehicle. Scale bar: 50 µm. N,O) Representative images of WB and quantitative analysis of pCREB expression for NP cells treated with CGRP for 0–120 min respectively. P,Q) Representative images of WB and quantitative analysis of CHSY1 expression for NP cells treated with CGRP, si‐RAMP1, CGRP+si‐RAMP1, CREB inhibitor 666‐15, and CGRP+666‐15, respectively. R) Quantitative analysis of CS by DMMB assay for NP cells treated with CGRP, si‐RAMP1, CGRP+si‐RAMP1, CREB inhibitor 666‐15, and CGRP + 666‐15, respectively. All data are presented as means ± SEM, *n* = 6 per group, *P < 0.05 and **P < 0.01 (A,C,D,F,O,Q,R). Statistical significance was determined by two‐tailed Student's *t*‐test. *P < 0.05 and **P < 0.01. Statistical significance was determined by one‐way ANOVA (H,J,K,M).

Sensory nerve also secretes several functional neuropeptides including vasoactive intestinal peptide (VIP), CGRP, neuropeptide Y (NPY), and substance P (SP).^[^ ^20^ ^]^ To further validate their effect on NP cells, we added these neuropeptides to stimulate mice NP cells. Intriguingly, only CGRP could significantly promote CS content and CHSY1 gene and protein expression as evidenced by RT‐PCR, WB, DMMB, and IF assays (**Figure** **5**A–F). Co‐culturing NP cells with sensory neuros indicated significant enhancement in NP cells CS production and CHSY1 gene expression compared with NP cells cultured alone, and this effect was obstructed by applying CGRP neutralizing antibody as evidenced by DMMB, RT‐PCR, western‐blot (WB), and immunofluorescence (IF) assay (Figure 5G–M). Mechanistically, we observed that CGRP could activate NP cell cyclic AMP response element‐binding protein (CREB) phosphorylation in a time‐dependent manner (Figure 5N,O). In addition, we also found that knockdown of receptor activity‐modifying protein 1 (RAMP1) by small interfering (si)‐RNA or CREB inhibitor 666‐15 application in NP cells could attenuate the increment of CHSY1 expression and CS production induced by CGRP treatment as evidenced by WB, RT‐PCR, and DMMB assays (Figure 5P–R). Taken together, these data indicate that CGRP could regulate NP cell CHSY1 expression via RAMP1/CREB signaling pathway.

### 2.6 Disrupted CGRP Production in DRG Induces IVD CS Reduction and IDD Phenotype

**Figure 6:**
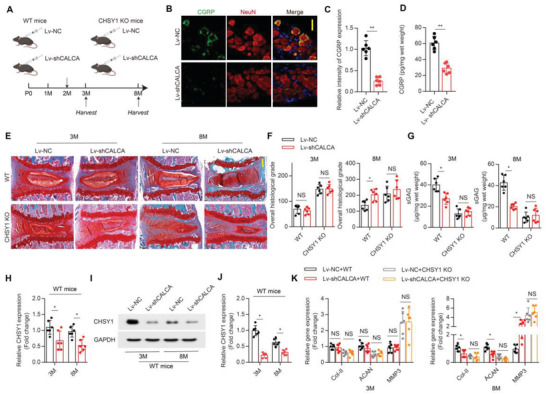
DRG CGRP secretion is essential for CS production and IVD homeostasis. A) Schematic graph of the injection of lentivirus packaged with short harpin (sh) CALCA gene (Lv‐shCALCA) or control vector (Lv‐NC). Male CHSY1 KO mice and their littermates were injected with Lv‐shCALCA or Lv‐NC at 2‐months‐old; mice were harvested at 3 and 8‐months‐old. B,C) Representative immunofluorescence co‐staining of CGRP (green) with NeuN (red) and quantitative analysis for L2 DRG from 3‐month‐old WT mice treated with Lv‐NC and Lv‐shCALCA. Scale bar: 10 µm. D) Quantitative analysis of ELISA assay for CGRP concentration in L5 lumbar disc from 3‐month‐old WT mice treated with Lv‐NC and Lv‐shCALCA. E,F) Representative images of SOFG and quantitative analysis of overall histological grade for 3 and 8‐month‐old CHSY1 KO mice and their littermates treated with Lv‐NC and Lv‐shCALCA. Scale bar: 50 µm. G) Quantitative analysis of CS by DMMB assay for NP derived from 3 and 8‐month‐old CHSY1 KO mice and their littermates treated with Lv‐NC and Lv‐shCALCA. H) Quantitative RT‐PCR analysis of CHSY1 expression of NP in 3 and 8‐month‐old WT mice treated with Lv‐NC and Lv‐shCALCA. I,J) Representative images of WB and quantitative analysis of CHSY1 expression of NP in 3 and 8‐month‐old WT mice treated with Lv‐NC and Lv‐shCALCA. K) Quantitative RT‐PCR analysis for NP Col‐II and ACAN and MMP3 expression from 3 and 8‐month‐old male CHSY1 KO and their littermates treated with Lv‐NC and Lv‐shCALCA. All data are presented as means ± SEM, *n* = 6 per group. **P < 0.01, statistical significance was determined by two‐tailed Student's *t*‐test (C,D). *P < 0.05, NS: not significant. Statistical significance was determined by one‐way ANOVA (H,J,K). *P < 0.05, NS: not significant. Statistical significance was determined by two‐way repeated measures ANOVA with Bonferroni post hoc test (F,G).

To further examine whether CGRP could regulate NP CS bio‐synthesis in vivo, we packaged short harpin (sh)‐CALCA RNA in lentivirus and intrathecally injected this vector or control vector into WT and CHSY1 KO mice to ablate DRG CGRP production (**Figure** **6**A). Four weeks after injection, CGRP level was significantly decreased in DRG neurons and NP tissue as evidenced by IF and ELISA assay (Figure 6B–D). Consistently, SOFG staining showed that knockdown of CGRP in DRG could induce significant IVDD phenotype in 8‐month‐old WT mice (Figure 6E,F); however, this effect was abolished in CHSY1 KO and 3‐month‐old WT mice (Figure 6E,F), suggesting CHSY1 mediates CGRP regulation on IVD homeostasis. Specifically, significant reduction of NP CS content and CHSY1 expression was observed both in 3 and 8‐month‐old WT mice after DRG CGRP knockdown as evidenced by DMMB, RT‐PCR, and WB assay (Figure 6G–J), while this effect was interrupted in CHSY1 KO mice (Figure 6G), indicating that rather than affecting IVDD progression in later adulthood, CGRP could rapidly affect NP CS synthesis in an earlier stage. Accordingly, elimination of CGRP in DRG could only decrease ECM anabolic genes (Col‐II and ACAN) and increase catabolic gene MMP3 expression in 8‐month‐old WT mice (Figure 6K).

### 2.7 Reduced RAMP1/CREB Signaling Correlates With Increased ECM Breakdown in Human Degenerated NP Sample

**Figure 7:**
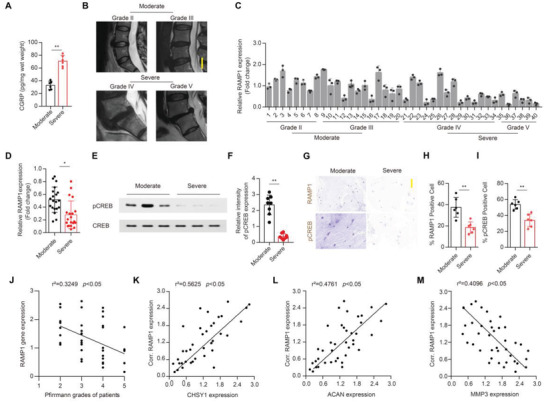
RAMP1/CREB signaling negatively correlates with ECM breakdown in human IVDD NP sample. A) Quantitative analysis of ELISA assay for CGRP concentration in human NP tissue under moderate and severe IVDD grade. B) Representative human lumbar spine MRI images for different IVDD grade ranging from Grade II to Grade V (Moderate: Grade II and Grade III, Severe: Grade IV and Grade V), scale bar: 2 cm. C,D) Quantitative RT‐PCR analysis for human NP tissue RAMP1 expression in different IVDD grade ranging from Grade II to Grade V (C) and of human NP tissue from moderate and severe IVDD grade patients (D). E,F) Representative images of WB and quantitative analysis of pCREB expression of human NP tissue from moderate and severe IVDD grade patients. G,H,I) Representative immunohistochemical staining and quantitative analysis of RAMP1 and pCREB expression in the human NP tissue from moderate and severe IVDD grade patients. Scale bar: 100 µm. J) Correlation analysis of relative RAMP1 expression in NP tissue and Pfirrmann grades of patients. K,L,M) Correlation analysis of relative RAMP1 expression in NP tissue and relative CHSY1 (K), ACAN (L), and MMP3 (M) expression. All data are presented as means ± SEM, *n* = 6 per group (A,H,I), *n* = 8 per group (F), *n* = 20 per group (D), *n* = 40 (J,K,L,M). *P < 0.05. **P < 0.01, statistical significance was determined by two‐tailed Student's *t*‐test (A,D,F,H,I). Statistical significance was determined by Pearson correlation analysis (J,K,L,M).

To testify CGRP regulation on NP cell CHSY1 expression clinically, we found that CGRP concentration was significantly higher in severe IVDD NP tissue relative to heathier NP samples (**Figure** **7**A,B), indicating uncoupled CGRP modulation on CHSY1 expression under human IVDD status. Intriguingly, we further found that RAMP1 gene expression and CREB activation were significantly decreased in degenerated NP samples as determined by RT‐PCR, WB, and IHC assay (Figure 7C–I), suggesting the CGRP signaling transduction was blocked due to decrement expression of RAMP1 under IVDD condition. Furthermore, we also showed that RAMP1 expression was significant negatively correlated with IVDD pfirrmann grades which represent disease severity (Figure 7J).^[^ ^21^ ^]^ In addition, correlation analysis revealed that RAMP1 expression was significant positively correlated with CHSY1 expression and ACAN expression, while negatively correlated with MMP3 expression in human NP tissue (Figure 7K–M). Overall, these data demonstrate that obstructed RAMP1/CREB signaling in IVDD might be responsible for loss of CHSY1 expression and disorganized NP ECM metabolism.

### 2.8 Activation of CREB Signaling by Forskolin Alleviates ECM Breakdown and IVDD Progression

**Figure 8:**
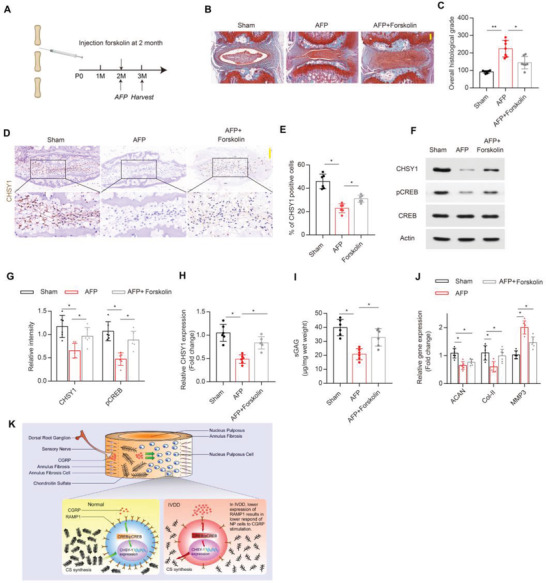
Forskolin alleviates ECM breakdown and IVDD via activation of CREB signaling. A) Schematic graph of the microinjection of 10 mg kg^−1^ forskolin into the mouse Co6‐Co7 coccygeal tail disc followed by AF puncture (AFP) IVDD model at 2‐month‐old and harvested one month later. B,C) Representative images of SOFG and quantitative analysis of overall histological grade for tail disc from male 3‐month‐old C57BL/6J that underwent sham, AFP, and AFP with forskolin treatment. Scale bar: 100 µm. D,E) Representative immunohistochemical staining and quantitative analysis of CHSY1 expression in tail disc from male 3‐month‐old C57BL/6J that underwent sham, AFP, and AFP with forskolin treatment. Scale bar: 100 µm. F,G) Representative images of WB and quantitative analysis of CHSY1 and pCREB expression for tail disc from male 3‐month‐old C57BL/6J that underwent sham, AFP, and AFP with forskolin treatment. H) Quantitative RT‐PCR analysis for CHSY1 expression of tail disc from male 3‐month‐old C57BL/6J that underwent sham, AFP, and AFP with forskolin treatment. I) Quantitative analysis of CS by DMMB assay for tail disc from male 3‐month‐old C57BL/6J that underwent sham, AFP, and AFP with forskolin treatment. J) Quantitative RT‐PCR analysis for ACAN, Col‐II, and MMP3 expression of tail disc from male 3‐month‐old C57BL/6J that underwent sham, AFP, and AFP with forskolin treatment. K) Diagram showing how sensory nerve maintains IVD ECM metabolism via CGRP/CHSY1 axis during IVD homeostasis and degeneration. All data are presented as means ± SEM, *n* = 6 per group, *P < 0.05 and **P < 0.01. Statistical significance was determined by one‐way ANOVA.

Several clinical trials demonstrated that a potent CREB activator forskolin could be beneficial for asthma, obesity, and inflammatory diseases treatment.^[^ ^22^, ^23^, ^24^ ^]^ To explore whether forskolin could aid IVDD progression by activating CREB signaling, we microinjected forskolin to NP site to induce enhancement of CREB signaling (**Figure** **8**A). Mice IVDD model was created by AF puncture (AFP) as previously reported.^[^ ^25^ ^]^ SOFG staining and magnetic resonance imaging (MRI) test showed AFP could significantly induce IVDD phenotype 4 weeks after the procedure, and this IVDD phenotype was significantly attenuated by forskolin treatment (Figure 8B,C; Figure S7A,B, Supporting Information), and this finding was in accordance with another line of evidence which revealed that RAMP1 overexpression in IVD alleviated AFP‐induced IVDD (Figure S8, Supporting Information), suggesting activation of RAMP1/CREB signaling ameliorates IVDD progression. Furthermore, consistent with previous results, we also observed significant reduction of CHSY1 expression, CS content, and CREB activation in AFP‐induced IVDD mice model (Figure 8D–I); however, these effects were abolished by forskolin intradiscal injection after AFP as evidenced by RT‐PCR, WB, IHC, and DMMB assays (Figure 8D–I). In addition, ECM anabolic genes Col‐II and ACAN expression were significantly increased, with ECM catabolic gene MMP3 expression decreased by forskolin treatment after AFP (Figure 8J). Graphic diagram indicates sensory nerve regulation on IVD ECM homeostasis under normal and IVDD conditions (Figure 8K). In summary, these data dictate the potential therapeutic role of CREB activator forskolin for IVDD treatment.

## 3 Discussion

Balanced ECM metabolism between anabolism and catabolism is essential for IVD homeostasis.^[^ ^10^, ^11^, ^26^ ^]^ Abundant studies have demonstrated the factors which could affect ECM metabolic activity, such as inflammatory cytokines, immune factors, and hypoxia.^[^ ^10^, ^11^, ^26^, ^27^ ^]^ However, these factors majorly play their roles in IVDD, while it is still elusive how normal IVD homeostasis is maintained. Sensory nerves are located at vertebrae between IVDs and outer layer of AF region under physiological conditions.^[^ ^28^ ^]^ Our previous study revealed that sensory nerve could regulate bone homeostasis via transmitting local prostaglandin E2 signaling to central nervous system to activate central regulation on bone density.^[^ ^9^, ^29^ ^]^ However, it is unknown whether there exists functional link between sensory nerve and IVD. It is possible that in IVD, microenvironment signals might trigger communications between IVD and central nervous system; it is also possible that sensory nerve could directly regulate IVD homeostasis locally via secreting sensory neuropeptides. In this study, we observed that ablation of CGRP local secreting capability of sensory nerve could induce IVDD in mice late adulthood; this is the first evidence indicating that sensory nerve could affect IVD ECM homeostasis via local secretory mechanisms. Intriguingly, our further experiments showed that sensory denervation also induced reduction of IVD water preservation molecule CS in mice in early adulthood, indicating this might trigger subsequent ECM breakdown that represents IVDD phenotype.

Though we observed sensory nerve affected CS synthesis in mice early adulthood, the structural integrity of IVD remained unaffected; this finding indicates that IVD at this temporal stage is actually an IVDD onset stage. Therefore, it is necessary to further analyze the underlying molecular mechanism; we found that CS biosynthesis family genes were down‐regulated by sensory denervation, especially for CHSY1 gene. By adopting CHSY1 KO mice, we observed reduction of CS content with normal IVD histologic feature in 1‐month‐old CHSY1 KO mice; these pieces of evidence dictate a temporal IVDD developing sequence that includes early CS reduction, IVD dehydration, then occurrence of IVDD hallmarks. In addition, this disease progression pattern is highly similar to clinical IVDD development.^[^ ^10^, ^11^ ^]^ Therefore, these observations highly indicate that CHSY1 KO‐induced CS reduction is hazardous to IVD homeostasis, which further triggers the IVDD pathological sequence.

Current knowledge reveals that sensory nerve could locally secrete diverse range of neuropeptides to affect organ functions, such as SP, CGRP, VIP, and NPY.^[^ ^20^ ^]^ Our results indicate that CGRP is the key molecule involved in sensory nerve regulation on NP CS metabolism. CGRP possesses a wide range of functions such as promoting bone formation and facilitating pain transduction;^[^ ^18^, ^30^ ^]^ it also serves as neurotransmitter in primary afferents.^[^ ^31^, ^32^ ^]^ To this end, we demonstrated CGRP regulated NP cell CHSY1 expression via RAMP1. RAMP1 acts as CGRP receptor component which can alter the sensitivity of cells to CGRP;^[^ ^33^ ^]^ therefore, low level of RAMP1 tunes down responsiveness of NP cells to CGRP; this explains why high level of CGRP is accompanied by low level of CHSY1 expression in NP tissue under IVDD conditions. Meanwhile, CGRP receptor is also a family B G‐protein‐coupled receptor (GPCR) which could further trigger cAMP activity and phosphorylation of CREB signaling.^[^ ^34^, ^35^, ^36^ ^]^ Indeed, we here showed that CGRP could also induce CREB phosphorylation and regulate CHSY1 expression via CREB signaling in NP cells. Our results also demonstrated that NPY could slightly increase NP cell CS production; however, we also showed that NPY could not affect CHSY1 expression, suggesting NPY regulation on NP cell CS synthesis was not through CHSY1 pathway.

Our study has some limitations. First, it is difficult to distinguish non‐degenerated IVDs from degenerated ones due to no gold standard diagnostic scale for IVDD that can be applied clinically. To this end, we applied the commonly used Pfirrmann grading system to cluster IVDs into different groups based on their disease severity, and indeed, we identified differences between the mild and severe IVDD groups. Next, sensory nerve releases other neuropeptide which might also possess functional role in NP cell regulation; for example, SP was known to promote IVD ECM degradation, therefore, we did not exclude the possibility that sensory nerve might hold bi‐regulation effect on IVD homeostasis, which required further investigation. In summary, our study first showed sensory nerve secretion of CGRP could promote NP CHSY1 expression via RAMP1/CREB signaling pathway; thus, to maintain IVD ECM homeostasis and promote IVD repair.

## 4 Conclusion

Our study first showed here that even without physical contact, sensory nerve secretion of CGRP could regulate NP CHSY1 expression via RAMP1/CREB signaling pathway; thus, to maintain IVD ECM homeostasis and promote IVD repair.

## 5 Experimental Section

### Animals and In Vivo Treatment

The *Advillin‐Cre* (*Avil‐Cre*) mouse strain was purchased from Shanghai Model Organisms Center. The *TrkA^fl/fl^* mice were obtained from Shen Liu (Shanghai Jiao Tong University). Heterozygous male *Avil‐Cre* mice (female *Avil‐Cre* mice were not used to breed in case of leakage of TRKA protein into the eggs) were crossed with a *TrkA^fl/fl^* mouse. The offspring were intercrossed to generate the following genotypes: wild type (referred to as “WT” in the text), *Avil‐Cre* (*Cre* recombinase expressed driven by *Advillin* promoter), and *Avil‐Cre::TrkA^fl/fl^* (referred to as “*TrkA_Avil_^–/–^*” in the text). To generate the inducible sensory denervation mouse model, 8‐week‐old WT mice were injected subcutaneously with capsaicin (30 mg per kg) four times a week for one week; to maintain denervation status, boosting injections were made every two months. CHSY1 knockout (KO) mice were generated by using CRISPR‐Cas9 technology with semi‐cloning technology as previously described.^[^ ^19^ ^]^ In brief, CRISPR‐Cas9 technology was introduced to generate CHSY1 KO artificial spermatids and then it was injected into the mature oocytes to obtain CHSY1^+/−^ female mice (F0). To generate CHSY1^−/−^ (CHSY1 KO) mice, the CHSY1^+/−^ females were backcrossed with wild‐type (WT) males (C57BL/6) to obtain CHSY1^+/−^ male and female mice (F1), which were then crossed to obtain CHSY1 KO mice (F2). The wild‐type littermates were used as controls. The detailed generation and verification procedure of CHSY1 KO mice is shown in Figure S1, Supporting Information. Six mice at a similar age were randomly selected and assigned to each group. All animals were maintained at the animal facility of the Naval Medical University. All experimental protocols were approved by the Animal Care and Use Committee of Shanghai Changzheng Hospital, Naval Medical University (approval number: 2021SL044).

### Collection and Grading of NP Tissue Samples

Informed consent was given by all participating patients to obtain human intervertebral tissue at surgery. The experimental methods were carried out in accordance with the approved guidelines and the study was authorized by the ethics committee of the Shanghai Changzheng Hospital, Naval Medical University (2021SL044). A total of 40 nucleus pulposus samples with different degrees of IVDD (*n* = 40; age 16 to 75 years, mean age 51.4 years) were obtained from patients (Table S1, Supporting Information), who underwent disc resection surgery or spinal fusion to relieve LBP. Patients diagnosed with classical sciatica were excluded from the experiment. MRI T‐2 weighted images were collected and the modified Pfirrmann grading system was used to evaluate the degree of IVDD. In this study, samples of Grade‐II and Grade‐III were referred to as the moderate IVDD group; Grade‐IV and Grade‐V samples were referred to as the severe IVDD group. For grading of mouse NP tissue, the previously described mouse NP tissue grading system was used;^[^ ^37^ ^]^ the detailed histological grading system is shown in Table S2, Supporting Information. The results were assessed independently by two senior diagnostic imaging technologists who were unaware of the treatment.

### Isolation of Mouse NP Cells

For cell extraction, NP tissue specimens were washed twice with PBS, then minced and digested with protease (2 U per mL) in DMEM/F12 medium (Gibco, Grand Island, NY, USA) for 30 min at 37 °C. NP cells were released from the NP tissues by treating with type II collagenase (Gibco, Cat. No. 17101‐015, 0.25 mg mL^−1^) for 4 h at 37 °C. The remaining cell suspension was transferred into a 40 µm cell strainer (BD Biosciences, Franklin Lakes, NJ, USA) and centrifuged at 800 × *g* for 5 min. The NP cells were resuspended in DMEM/F12 containing 10% FBS (Gibco), penicillin (100 U per mL), streptomycin (100 µg per mL), and 1% L‐glutamine. The viability of the suspended cells was over 90% when assessed using cell counting kit‐8 (Dojindo, Tokyo, Japan). Cells were incubated at 37 °C in 5% CO2 and the medium was changed every 3 days. Cells at the second passage were used for subsequent experimental procedures.

### DRG Cell Extraction

DRGs from the L2–L5 spinal levels of 8‐week‐old mice were isolated in cold DMEM/F12 medium (Invitrogen, 11039‐021) and then treated with collagenase type A (Roche, 10103578001) at 37 °C. After trituration and centrifugation, cells were resuspended and plated on glass coverslips coated with ploy‐D‐lysine and laminin. The cells were then cultured in an incubator at 37 °C.

### Si‐RNA Transfection

Pre‐designed si‐RNA and negative control (NC) si‐RNA to silence the murine RAMP1 gene were purchased from the Genepharma Company (Shanghai, China). For transfection, NP cells were seeded into 6‐well plates, incubated for 24 h, then transfected with RAMP1 si‐RNA (100 nm) or negative control si‐RNA, using Lipofectamine RNAiMAX Transfection Reagent (Invitrogen, 13778150) according to the manufacturer's instructions. After subsequent treatments, cells were harvested for analysis.

### Quantitative Real‐Time Polymerase Reaction Chain (qPCR)

Total RNA was purified from cells in culture or tissues using TRIzol (Invitrogen, 15596026), following the manufacturer's protocol. qPCR was performed using the Taq SYBR Green Power PCR Master Mix (Invitrogen, A25777) on a Step One Plus real‐time PCR system (Applied biosystems, Foster City, CA, USA); GAPDH amplification was used as an internal control. Dissociation curve analysis was performed for every experiment. Sequences of the primers used for each gene are available on request.

### DMMB Assay

The DMMB (1,9‐dimethylmethylene blue) assay was performed using a Blyscan sGAG assay kit (Biocolor, B1000, Biocolor, Carrickfergus, UK) according to the manufacturer's instructions. Briefly, harvested NP tissues or NP cells were washed with PBS and digested with 1 mL papain extraction reagent for 24 h at 65 °C. CS contents were determined by reaction with DMMB, and staining was quantified by measuring absorbance at 656 nm. Chondroitin‐4‐sulfate was used as the standard.

### Enzyme‐Linked Immunosorbent Assay

NP tissue from both mice and human were collected for evaluation of CGRP content. The NP tissue was digested using T‐PER buffer (Thermo Scientific, 78510); the extracted proteins were quantified by using an ELISA kit (Novus biologicals, NBP2‐75259) according to the manufacturer's instructions.

### Western Blot Analysis

NP tissue and cells were harvested using ReadyPrep Protein Extraction Kit (Bio‐Rad, 1632086). Protein concentration was determined using a bicinchoninic acid protein assay kit (Pierce Biotechnology, 23225). Immunolabeling was detected using the Pierce ECL western blotting substrate (Pierce Biotechnology, 32132). The following antibodies and dilutions were used: CHSY1 antibody (1:1000) (Proteintech, 14420‐1‐AP), GAPDH (1:2000) (Abcam, ab9485); phospho‐CREB (1:1000) (Abcam, ab32096), CREB (1:1000) (Abcam, ab32515), RAMP1 (1:1000) (Abcam, ab156575).

### Immunocytochemistry

Murine NP cells were plated into 24‐well plates and treated in various conditions as described in the figure legends. After incubation, the cells were fixed with 4% paraformaldehyde and permeabilized with 0.3% Triton X‐100 in PBS for 10 min. Next, the cells were blocked with 5% bovine serum albumin in PBS and incubated with a mouse monoclonal antibody against chondroitin sulfate (1:200 dilution) (Sigma–Aldrich, C8035) at 4 °C overnight. The cells were washed with PBS and incubated with the secondary antibody goat anti‐mouse IgG‐FITC (1:128 dilution) (Sigma–Aldrich; F5262) for 1 h at room temperature. Samples were imaged using a fluorescence microscope (Nikon, Ni‐E, Japan). The average relative fluorescence intensity of each cell was analyzed using the image J software (NIH, Bethesda, MD, USA)

### Label‐Free Proteomic Profiling and Bioinformatic Analysis

The supernatant of DRG cells was collected every 2 days with regular culture medium. The protein contents of the supernatants were concentrated using Amicon Ultra filters (10 kDa cut‐off, UFC9010, Millipore, USA). Trichloroacetic acid (TCA) was used to precipitate proteins for 30 min at 4 °C and centrifuged at 40 000 × g for 30 min. The concentration was measured according to the manufacturer's instructions using Qubit quantification kit (Invitrogen, CA, USA) to determine the concentration. Label‐free proteomic profiling of the protein extracts was done by Beijing BangFei Bioscience Co., Ltd. In brief, protein samples (60 µg) were used and addition of DTT (DL‐Dithiothreitol, 100 mmol L^−1^) to its final concentration (10 mmol L^−1^) was performed and mixed at 37 °C for 60 min. The mix was then diluted with IAM (Iodoacetamide, 250 mmol L^−1^) and kept in the dark for 60 min. UA buffer (8 m urea, 100 mm Tris‐HCl, pH 8.0, 100 µL) was used to wash the samples for two times, and followed by three washes with NH_4_HCO_3_ (50 mm, 100 µL). Last, samples were digested with trypsin for 12 h at 37 °C, and the supernatant was loaded onto a silica capillary column packed with 3‐µm dionex C18 material. Agilent 1100 quaternary HPLC (high performance liquid chromatography) was used to analyze the sample using 12‐step separation after desalting. Mass spectrometer was then operated in the MS/MS (auto) mode to acquire the raw data. The data were further processed and expressed proteins with *P* < 0.05 and signal > 1.5‐fold were considered as differentially expressed proteins. The processed log2‐transformed and quantile‐normalized signal data were used, and genes with *P* < 0.05, signal fold‐change ≥ ±1.5 were filtered and considered as differentially expressed genes. Unsupervised hierarchical clustering was performed to detect the expression pattern change using the profiling data by average linkage and median centering; the processed data was visualized with TreeView. Other analysis like Gene Ontology (GO) analysis was carried out using annotations in PANTHER database v 6.1 (www.pantherdb.org), and pathway analysis was performed with the tools on the KEGG database (http://www.genome.jp/kegg/pathway.html).

### Intrathecal Lentivirus Vector Injection

Following a small skin incision along the lower lumbar spine level to visualize the spine, the lentiviral vector was delivered into the L2–L3 intervertebral space of anesthetized mice as described previously.^[^ ^38^ ^]^ In brief, a Hamilton syringe (100‐µL) connected to a 30G needle was used to inject lentiviral stock (30 µL) containing an estimated 9 × 10^8^ (Lv‐CALCA) and 1 × 10^10^ (Lv‐NC) transducing units (TU) per mL. Appropriate access to the intrathecal space was confirmed by animal's tail movement. Lentiviral vector was constructed by the Genepharma Company (Shanghai, China); briefly, the sh‐CALCA and NC sequence were recombined with lentiviral vectors, After DNA sequencing identification, the shRNA recombinant vectors were co‐transfected into 293T cells with pGag/Pol, pRev, and pVSV‐G to packaging lentiviral particles and the virus titers were determined. The shRNA sequence targeting murine CALCA was 5′‐GCACGTACACACAAGACCT‐3′.

### AFP Procedure

The IVDD model was generated in 8‐week‐old WT C57BL/6 mice by the AF needle puncture as previously described.^[^ ^25^ ^]^ In brief, mice underwent general anesthesia. A sagittal small skin incision was performed from Co6 to Co8 to help locate the disc position for needle insertion in the tail. Subsequently, Co6–Co7 coccygeal discs were punctured using a syringe needle. The syringe needle was inserted into Co6–Co7 disc along vertical direction and then rotated in the axial direction by 180° and held for 10 s. The puncture was made parallel to the endplates through the AF into the NP using a 31‐G needle, which was inserted 1.5 mm into the disc to depressurize the nucleus. The other segments were left undisturbed as a contrast segment. IVDs were harvested at 6 weeks post‐surgery. All procedures and protocols were approved by the ethics committee of Naval Medical University.

### Immunohistochemistry, Immunofluorescence Assay

Human NP tissue, mice lumbar spine, and DRGs were collected and fixed in 4% paraformaldehyde for 24 h or overnight. Then, the spine samples were decalcified in EDTA (pH 7.4, 10% or 0.5 m) for 21 or 5 days and embedded in paraffin or optimal cutting temperature compound (Sakura Finetek). 4‐µm‐thick sagittal‐oriented sections of the lumbar spine were processed for SOFG and immunohistochemical staining using a standard protocol. 40‐µm‐thick lumbar spine sections were prepared for sensory nerve‐related immunofluorescent staining and 20‐µm‐thick DRG sections were prepared for sensory neuro‐related immunofluorescent staining by using a standard protocol. The sections were incubated with primary antibodies to mouse CGRP (1:100, ab81887, Abcam), CHSY1 (1:100, 14420‐1‐AP, Proteintech), phospho‐CREB (1:100, ab32096, Abcam), RAMP1 (1:200, ab203282, Abcam), and NeuN (1:500, MAB377, Sigma) overnight at 4  °C. Then, the corresponding secondary antibodies were added onto the sections for 1  h while avoiding light. For immunohistochemistry, a streptavidin horseradish peroxidase detection kit (Biolegend) was subsequently used to detect the immunoactivity, followed by counterstaining with hematoxylin (Sigma). For immunofluorescent staining, the sections were counterstained with 4′,6‐diamidino‐2‐phenylindole (DAPI, Invitrogen). The sample images were observed and captured by a fluorescence microscope (Nikon, Ni‐E, Japan) or confocal microscope (Zeiss LSM 780). ImageJ (NIH) software was used for quantitative analysis.

### Statistical Analysis

Statistical analyses were performed with GraphPad Prism 9.0 software (GraphPad Software, Inc., San Diego, CA, USA). Data were presented as means ± standard error of mean (SEM) of at least three independent experiments. For comparisons between two groups, two‐tailed Student's *t* tests were used. For comparisons among multiple groups, two‐way repeated measures analysis of variance (ANOVA) with Bonferroni post hoc test was used. *P* values < 0.05 were considered significant. The statistical analyses and sample size applied for each experiment are indicated in the figure legends.

## Conflict of Interest

The authors declare no conflict of interest.

## Supporting information

Supporting Information

## Data Availability

The data that support the findings of this study are available from the corresponding author upon reasonable request.
