# Supplementary material for: Sensory Nerve Maintains Intervertebral Disc Extracellular Matrix Homeostasis Via CGRP/CHSY1 Axis
Source: Adv Sci (Weinh). 2022 Sep 1;9(30):2202620. doi: 10.1002/advs.202202620 (PMC9596848; doi:10.1002/advs.202202620)
Supplement: Supplementary file 1 — Supporting Information [file ADVS-9-2202620-s001.pdf]

ADVANCED SCIENCE
Open Access

Supporting Information

for *Adv. Sci.*, DOI 10.1002/advs.202202620

Sensory Nerve Maintains Intervertebral Disc Extracellular Matrix Homeostasis Via CGRP/CHSY1 Axis

Bo Hu, Xiao Lv, Leixin Wei, Yunhao Wang, Genjiang Zheng, Chen Yang, Fazhi Zang, Jianxi Wang, Jing Li, Xiaodong Wu, Zhihao Yue, Qiangqiang Xiao, Zengwu Shao, Wen Yuan, Jinsong Li, Peng Cao, Chen Xu* and Huajiang Chen**

Supporting Information

Sensory nerve maintains intervertebral disc extracellular matrix homeostasis via CGRP/CHSY1 axis

Bo Hu, Xiao Lv, Leixin Wei, Yunhao Wang, Genjiang Zheng, Chen Yang, Fazhi Zang, Jianxi Wang, Jing Li, Xiaodong Wu, Zhihao Yue, Qiangqiang Xiao, Zengwu Shao, Wen Yuan, Jinsong Li, Peng Cao, Chen Xu* and Huajiang Chen**

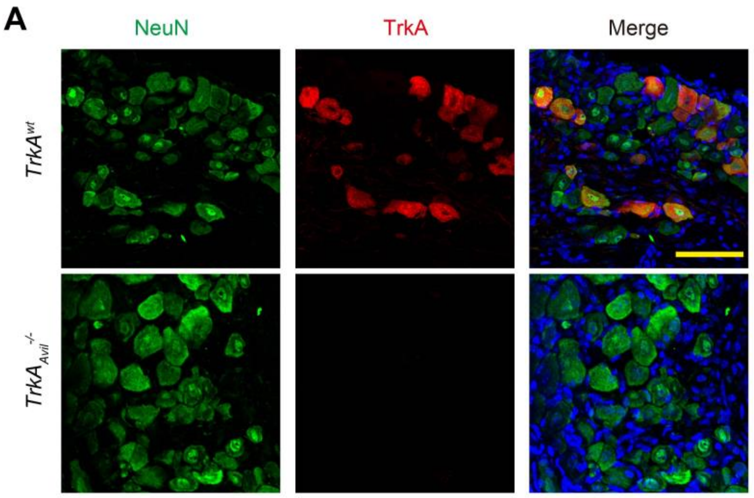

Figure S1. Knockout efficiency of genetical sensory denervation model. A) Representative images of immunohistochemical staining for NeuN (Green) and TrkA (Red) and co-staining for NeuN (Green) and TrkA (Red) for the Dorsal Root Ganglion (DRG) of 3-month-old male $TrkA^{wt}$ and $TrkA_{Avil}^{-/-}$ mice. Scale bar :50 μm.

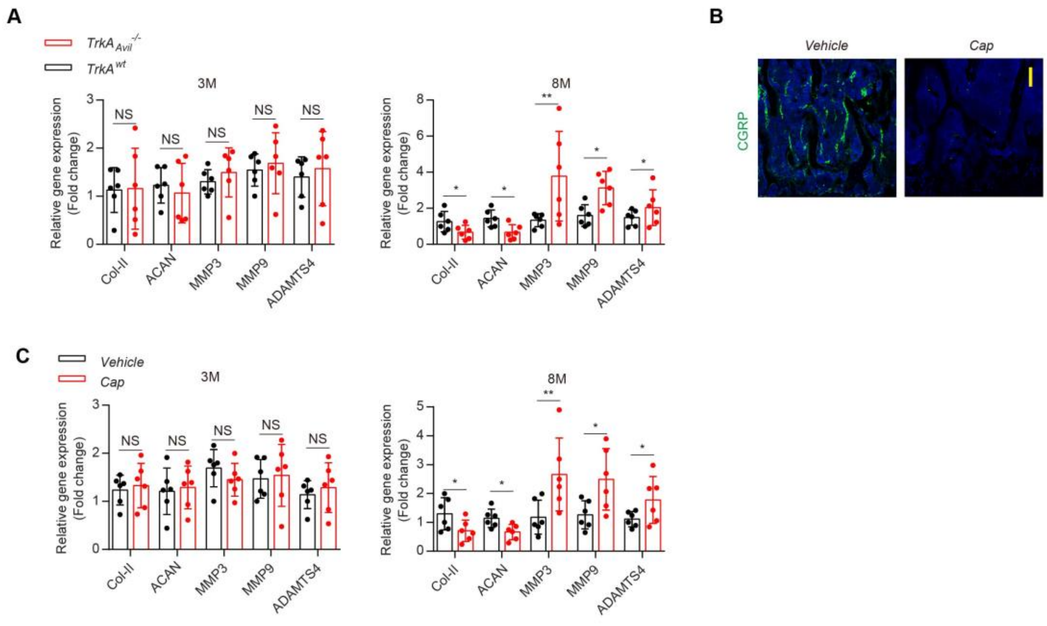

Figure S2. Sensory denervation induced ECM metabolic disorder in IVD. A) Quantitative RT-PCR analysis nucleus pulposus (NP) extracellular extracellular matrix (ECM) anabolic (Col-II and ACAN) and catabolic (MMP3, MMP9, ADAMTs4) relative genes expression from 3 and 8-month-old male $TrkA^{wt}$ and $TrkA_{Avil}^{-/-}$ mice. B) Representative immunofluorescence staining of $CGRP^{+}$ sensory

nerves (green) in the vertebra from 3-month-old male C57B6/J mice treated with capsaicin and vehicle. Scale bar: 100µm. C) Quantitative RT-PCR analysis nucleus pulposus (NP) extracellular extracellular matrix (ECM) anabolic (Col-II and ACAN) and catabolic (MMP3, MMP9, ADAMTs4) relative genes expression from 3 and 8-month-old male C57B6/J mice treated with capsaicin and vehicle. All data are presented as means ± SEM, n = 6 per group, *P<0.05, **P<0.01, NS: not significant. Statistical significance was determined by two-tailed Student's t-test.

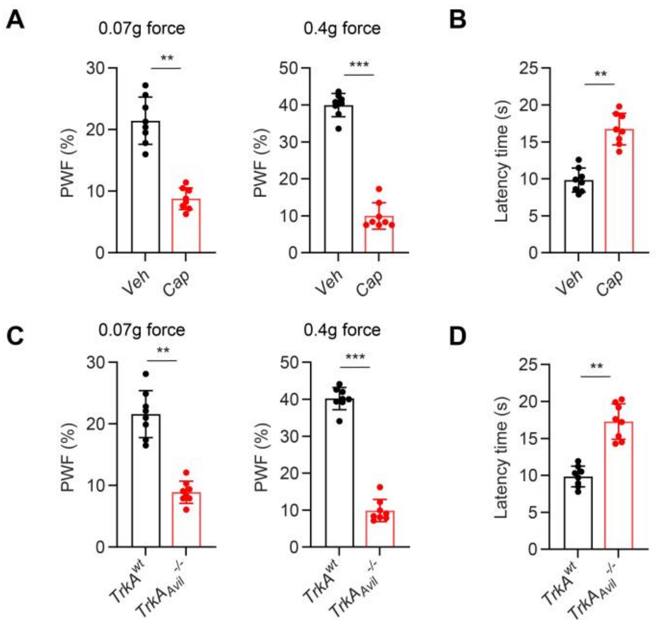

Figure S3. Pain sensitivity was reduced after sensory denervation. A) Quantitative analysis of the frequency of paw withdrawal in response to application of 0.07 and 0.4g force via a von Frey filament of 3-month-old WT male mice injected with vehicle or capsaicin (30 mg/kg per day). B) Quantitative analysis of the latency time for 3-month-old male WT male mice injected with vehicle or capsaicin (30 mg/kg per day) in hotplate test. C) Quantitative analysis of the frequency of paw withdrawal in response to application of 0.07 and 0.4g force via a von Frey filament of 3-month-old male $TrkA^{wt}$ and $TrkA_{Avil}^{-/-}$ mice. D) Quantitative analysis of the latency time for 3-month-old male $TrkA^{wt}$ and $TrkA_{Avil}^{-/-}$ mice. The latency time was defined as the time to the first sign of paw licking or jumping response. All data are presented as

means ± SEM, *n* =8 per group, **P*<0.05, ***P*<0.01, *** *P*<0.001. Statistical significance was determined by two-tailed Student’s t-test.

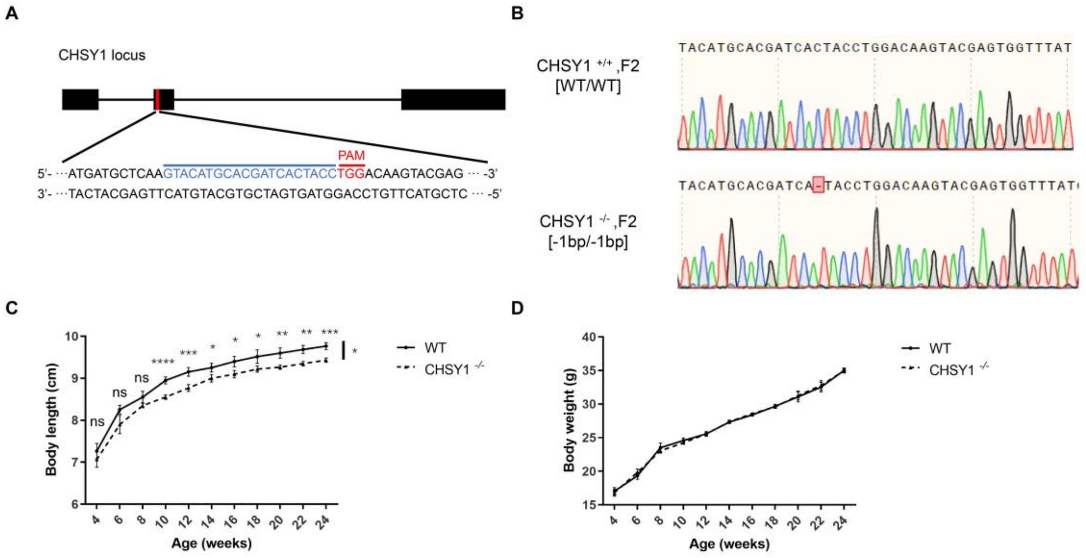

Figure S4. Efficient generation of CHSY1 KO mice. A) Schematic diagram of CHSY1 sgRNA design. The red bases represent the PAM sequence, while the blue bases represent sequence of *CHSY1* sgRNA. B) Genotyping of CHSY1 F2 mice. C) Dynamic change of body length between WT and CHSY1$^{-/-}$ mice. n = 6 per group. D) Dynamic change of body weight between WT and CHSY1$^{-/-}$ mice. n = 6 per group. All data are shown as mean ± SEM. *P < 0.05, **P < 0.01, ***P < 0.001, ****P < 0.0001. Statistical significance was determined by two-tailed Student’s t-test.

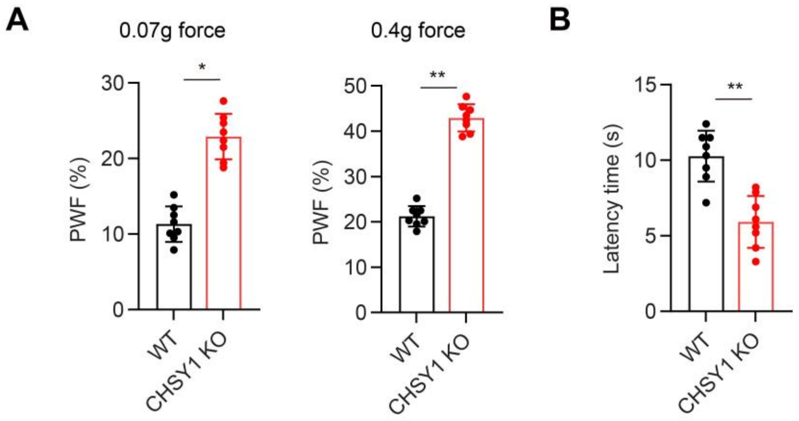

Figure S5. Pain sensitivity was increased in CHSY1 KO mice model. A) Quantitative analysis of the frequency of paw withdrawal in response to application of 0.07 and 0.4g force via a von Frey filament of 3-month-old male WT and CHSY1 KO mice. B) Quantitative analysis of the latency time for 3-month-old male WT and CHSY1 KO mice. The latency time was defined as the time to the first sign of paw licking or jumping response. All data are presented as means ± SEM, n=8 per group, *P<0.05, **P<0.01. Statistical significance was determined by two-tailed Student's t-test.

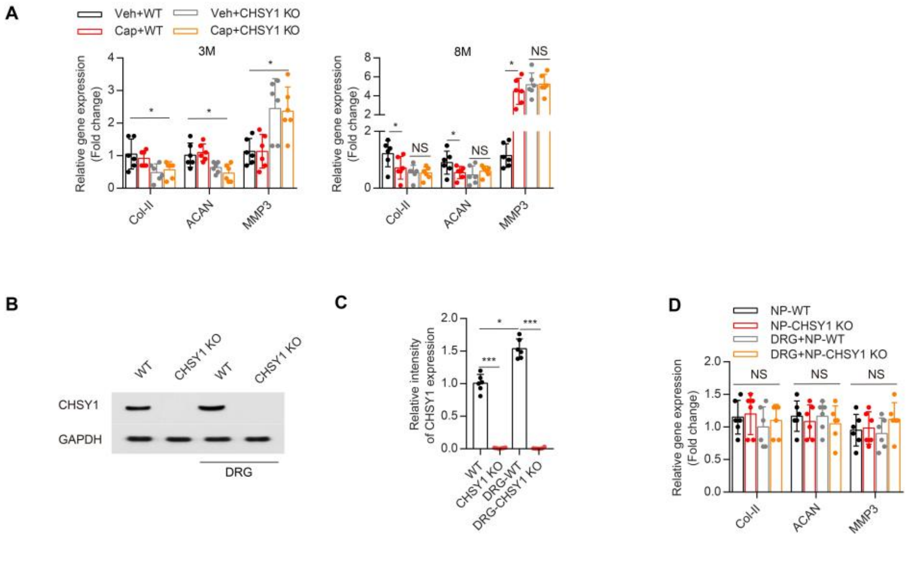

Figure S6. Sensory nerve regulation on IVD ECM metabolism was through CHSY1 signaling. A) Quantitative RT-PCR analysis of NP Col-II and AGG and MMP3 expression from 3 and 8-month-old male CHSY1 KO and their littermates

treated with vehicle and capsaicin. B, C) Representative images of Western blot (WB) and quantitative analysis of CHSY1 expression for NP cells which from CHSY1 KO mice and littermates, cultured with DRG neurons or cultured alone. D) Quantitative RT-PCR analysis of Col-II and AGG and MMP3 expression in NP cells from CHSY1 KO mice and littermates, cultured with DRG neurons or cultured alone. All data are presented as means ± SEM, *n* = 6 per group, A, D) *P<0.05, NS: not significant. Statistical significance was determined by one-way ANOVA. C) *P<0.05, *** P<0.001. Statistical significance was determined by two-way repeated measures ANOVA with Bonferroni post hoc test.

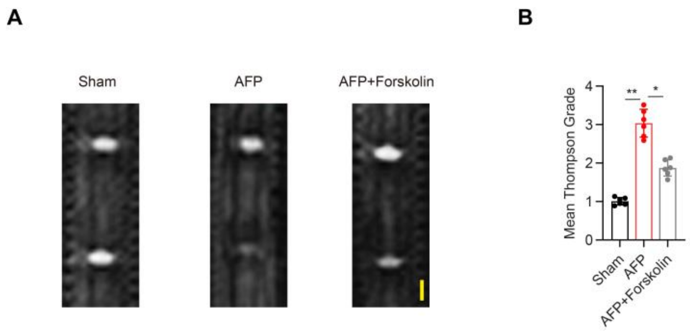

Figure S7. Forskolin alleviates loss of water content of IVD after AFP. A) Representative sagittal MRI T2-weighted images of the tail intervertebral disc for from 3-month-old male C57BL/6J underwent sham, AFP and AFP with one-month-forskolin treatment, scale bar: 500μm. B) Thompson grade of the tail disc for from 3-month-old male C57BL/6J underwent sham, AFP and AFP with one-month-forskolin treatment. All data are presented as means ± SEM, *n* = 6 per group, *P<0.05, **P<0.01. Statistical significance was determined by two-tailed Student's t-test.

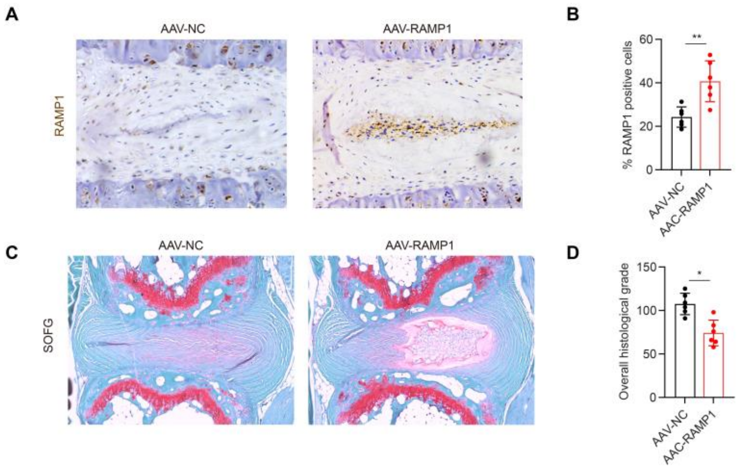

Figure S8. Overexpression of RAMP1 in IVD attenuates IVDD progression. A, B) 1×10^{10} RAMP1 overexpression vector packed in adeno-associated virus (AAV-RAMP1) in a volume of 10 μL was injection into the WT mice disc in AFP model at 2-month-old, negative control AAV vector (AAV-NC) was used as controls. Representative immunohistochemical staining of RAMP1 and quantitative analysis of RAMP1 expression in tail disc from 3-month-old WT mice treated with AAV-NC and AAV-RAMP1. C, D) Representative images of SOFG staining and quantitative analysis in tail disc from 3-month-old WT mice treated with AAV-NC and AAV-RAMP1. All data are means ± SEM, n = 6 per group, *P<0.05, **P<0.01. Statistical significance was determined by two-tailed Student's t-test.

Table S1. Patients' information

laboratory number	age	sex	disc level	MRI diagnosis	Pfirrmann grade	duration of symptoms (mo)
1	37	M	L4/5	relatively normal	2	5
2	28	M	L5/S1	relatively normal	2	11
3	39	M	L4/5	relatively normal	2	21
4	35	M	L4/5	relatively normal	2	26
5	38	M	L4/5	relatively normal	2	32
6	16	M	L3/4	relatively normal	2	9
7	33	M	L4/5	relatively normal	2	8
8	36	M	L5/S1	relatively normal	2	5
9	71	M	L3/4	mild degeneration	3	7
10	32	M	L5/S1	mild degeneration	3	27
11	63	M	L3/4	mild degeneration	3	18
12	36	M	L3/4	mild degeneration	3	11
13	48	M	L4/5	mild degeneration	3	20
14	49	M	L5/S1	mild degeneration	3	17
15	34	M	L5/S1	mild degeneration	3	30
16	72	M	L3/4	mild degeneration	3	15
17	58	M	L4/5	mild degeneration	3	27
18	68	M	L5/S1	mild degeneration	3	13
19	40	M	L4/5	mild degeneration	3	28
20	32	M	L4/5	mild degeneration	3	15
21	55	M	L5/S1	degenerate	4	18
22	61	M	L5/S1	degenerate	4	17
23	59	M	L4/5	degenerate	4	32
24	56	M	L4/5	degenerate	4	25
25	62	M	L5/S1	degenerate	4	18
26	72	M	L4/5	degenerate	4	14
27	53	M	L3/4	degenerate	4	31
28	59	M	L3/4	degenerate	4	15
29	50	M	L5/S1	degenerate	4	23
30	54	M	L5/S1	degenerate	4	16
31	72	M	L4/5	degenerate	4	26
32	53	M	L4/5	degenerate	4	27
33	61	M	L5/S1	degenerate	4	26
34	56	M	L4/5	degenerate	4	4
35	42	M	L5/S1	severe degeneration	5	32
36	58	M	L4/5	severe degeneration	5	32
37	62	M	L5/S1	severe degeneration	5	28
38	61	M	L3/4	severe degeneration	5	21
39	75	M	L4/5	severe degeneration	5	17
40	70	M	L3/4	severe degeneration	5	24

Table S2. Histological grading system

Category	Description
Organization of the Annulus Fibrosus	0=normal; organized lamellar structure<br>25=mild serpentine patterning<br>50=mild in-folding and disorganization of lamellae<br>75=moderate disorganization of lamellae<br>100=severe disorganization and in-folding of lamellae
Annulus Fibrosus/Nucleus Pulposus Border	0=clear border between the AF and NP<br>50=loss of distinction between the AF and np<br>100=border between the AF and NP not discernable
Nucleus Pulposus Extracellular Matrix	0=gelatinous, proteoglycan rich NP ECM<br>25 = mild condensation or fragmentation of NP ECM or mild reduction in glycosaminoglycan staining<br>50=moderate condensation of NP ECM or moderate reduction in glycosaminoglycan staining<br>75=severe NP ECM condensation or fragmentation or severe reduction in glycosaminoglycan staining<br>100=Complete absence of glycosaminoglycan staining, marked fibrosis of NP
Nucleus Pulposus Cellularity	0=many notochordal/chondrocyte-like cells<br>25=mild reduction in notochordal/chondrocyte-like cell populations<br>50=significant reduction in chondrocyte-like cells<br>75=rare chondrocyte-like cells, evidence of early fibrosis<br>100=fibroblast-like cells with surrounding fibrosis, absence of any chondrocyte-like cells
